# Risk factors for perimenopausal depression in Chinese women: a meta-analysis

**DOI:** 10.3389/fpsyt.2023.1199806

**Published:** 2023-10-11

**Authors:** Qingwen Gan, Ran Yu, Zerong Lian, Lihua Wei, Yuanping Li, Yiling Yuan, Lilan Zheng

**Affiliations:** ^1^School of Nursing, Nanchang University, Nanchang, China; ^2^The First Affiliated Hospital of Nanchang University, Nanchang, China; ^3^Heping Hospital Affiliated to Changzhi Medical College, Changzhi, China

**Keywords:** perimenopausal women, depression, risk factors, China, meta-analysis

## Abstract

**Objective:**

To systematically evaluate the risk factors for perimenopausal depression in Chinese women and to provide a basis for screening perimenopausal women at high-risk for depression.

**Methods:**

A computer search of seven databases, including SinoMed, PubMed, Web of Science, and so on, and two clinical trial registries on the risk factors for depression in Chinese women during perimenopause was conducted for meta-analysis. The search time limit was from the establishment of the database to December 2022. The included case–control and cross-sectional studies were evaluated using the Newcastle–Ottawa scale (NOS) and criteria developed by the Agency for Healthcare Research and Quality (AHRQ).

**Results:**

A total of 15 papers with 12,168 patients and 18 risk factors were included. Meta-analysis results showed that the risk factors for depression in perimenopausal women were relationship quality [OR = 1.23, 95% confidence intervals (1.03, 1.46)], marital status [OR = 2.49, 95% CI (1.77, 3.50)], family income [OR = 1.48 95% CI (1.10, 2.00)], comorbid chronic diseases [OR = 2.39, 95% CI (1.93, 2.95)], exercise status [OR = 1.63, 95% CI (1.26, 2.11)], perimenopausal syndrome [OR = 2.36, 95% CI (2.11, 2.63)], age [OR = 1.04, 95% CI (1.01, 1.07)], and stressful events [OR = 12.14, 95% CI (6.48, 22.72)], and social support was a protective factor [OR = 0.76, 95% CI (0.63, 0.91), *p* < 0.05].

**Conclusion:**

Based on the exploration of risk factors for perimenopausal depression in Chinese women, we aimed to provide guidance for the screening of risk factors for depression in perimenopausal women and thereby reduce the incidence of depression.

**Systematic review registration:**

https://www.crd.york.ac.uk/PROSPERO/#myprospero, CRD42023403972.

## 1. Introduction

Perimenopause is an important stage for women that marks the transition from the reproductive phase to older age and is an important demarcation line for psychological and physiological changes in women. It is estimated that the number of perimenopausal women will reach 1.2 billion worldwide in 2030, including more than 280 million perimenopausal women in China (1). Perimenopausal women not only suffer from the physical discomfort brought by perimenopausal syndrome but also face enormous pressure from society, work, and family (2), and according to the latest report of the International Study of Mental Health on Women’s Health, perimenopausal or postmenopausal women are three times more likely to experience depression than premenopausal or menstruating women (3). Perimenopausal depression is a prominent public health problem that seriously affects the quality of life and sleep status of middle-aged women (4) and results in a series of health problems, such as cognitive decline, cardiovascular disease, osteoporosis, and sexual dysfunction (5, 6). It has been reported that depression is closely associated with suicidal tendencies (7, 8), with 26.5% of adults with major depression attempting suicide, causing serious distress to society and families. Therefore, the early identification of risk factors for depression and implementation of suitable interventions for perimenopausal women is the key to effectively reducing the incidence of depression in this group of individuals.

Currently, most of the studies on risk factors for depression in Chinese perimenopausal women are single studies that are influenced by sample size, study population, and study site, and the results regarding perimenopausal depression risk factors are still controversial. Therefore, this study systematically evaluated the literature on risk factors for perimenopausal depression in Chinese women based on clinical questions with the aim of providing evidence-based guidance for the screening of risk factors for depression in perimenopausal women worldwide, thus providing a basis for early identification and prevention of depression in perimenopausal women.

## 2. Methods

### 2.1. Study profile

This study has been registered on the PROSPERO platform as required under registration number CRD42023403972.

### 2.2. Search strategy

The literature search and pooled analysis were performed according to the latest version of the Preferred Reporting Item for Systematic Reviews and Meta-Analyses (PRISMA) statement (9). Computer searches were conducted in the China National Knowledge Infrastructure (CNKI), Wanfang database, Weipu (VIP), SinoMed, PubMed, Web of Science, and the Cochrane Library databases and in 2 clinical trial registries (ClinicalTrials.gov and Chinese Clinical Trail Registry) about studies on risk factors for perimenopausal depression in Chinese women. The search time limit was from the establishment of the database to December 2022. The final search strategy was determined after an initial search by 2 researchers (first and third author) using an advanced search strategy; the search terms included 3 main core elements: (a) menopause (e.g., perimenopause, perimenopausal syndrome, climacteric, menopausal syndrome); (b) depression (e.g., depressive symptoms, depressive symptoms, emotional depression); and (c) risk factors (e.g., risk factors, related factors, influence factor, factor risk). Detailed retrieval strategies are available as Supplementary material in the submission system at Frontiers – Submission.^1^

### 2.3. Inclusion and exclusion criteria

Inclusion criteria were as follows: (a) the study population was perimenopausal Chinese women (1 year from the start of menstrual disorders to menopause); (b) the study was an epidemiological study of risk factors for perimenopausal depression in Chinese women; (c) the outcome indicators were perimenopausal Chinese women who presented with depression, and OR values and 95% CI or convertible data were provided; and (d) the type of study was either a cross-sectional study or a case–control study.

Exclusion criteria were as follows: (a) duplicate publications; (b) reviews, systematic evaluations, and conference papers; (c) literature for which valid data could not be extracted; and (d) literature of low quality.

### 2.4. Literature screening and data extraction

Two investigators (first and second author) imported the included studies into EndNote literature management software, used the EndNote automatic check function and manually removed duplicate literature, read the titles and abstracts of the literature for initial screening, excluded literature with inconsistent study content and study design, and read the full text of the remaining literature to determine the final included literature. Two investigators extracted the basic information of the included literature, including first author, year of publication, study type, region, sample size, risk factors, OR values, and 95% CI. If there were differences at any point throughout the entire process of document selection and data extraction, the two researchers discussed the difference and negotiated, and if there was any disagreement, the corresponding author was consulted.

### 2.5. Quality assessment

The appropriate literature quality evaluation tools were used according to the different types of literature, and the quality of the included case–control studies was evaluated using the Newcastle–Ottawa scale (NOS) (9), and those with scores <5 were considered low-quality studies. The US-based Agency for Healthcare Research and Quality (AHRQ) criteria were used to evaluate the quality of the included cross-sectional studies (10); studies with scores <6 were considered low-quality studies, and low-quality studies were excluded from this study.

### 2.6. Statistical analysis

Data analysis was performed using Reviewer Manager 5.4 software. The heterogeneity of the included studies was determined by the *Q* test and *I*^2^, and when *p* > 0.1 and *I*^2^ ≤ 50%, the heterogeneity was small, and the fixed-effects model was used for data analysis; when *p* < 0.1 or *I*^2^ > 50%, statistical heterogeneity existed, and the random-effects model was used for data analysis. The stability of the meta-analysis results was tested by sensitivity analysis through a piecewise exclusion method and differences in the combined effect sizes of the two effect models; funnel plots and Egger′s test were used to determine whether the included studies had publication bias (when Egger′s test *p* > 0.05, there was no publication bias).

## 3. Results

### 3.1. Search results

**Figure 1:**
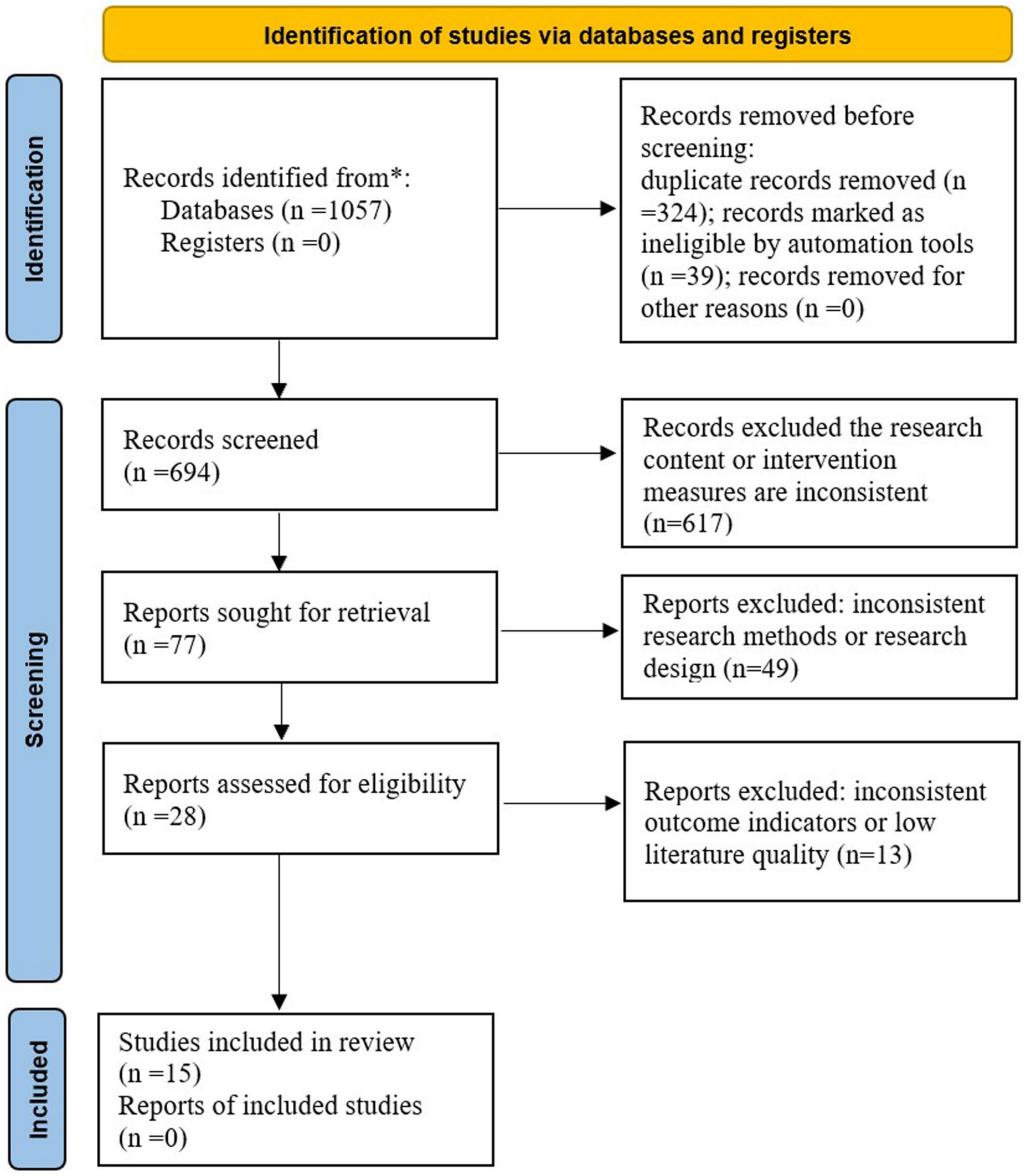
Diagram of the literature selection process.

A total of 1,057 studies were retrieved, including 770 Chinese studies and 287 English studies; 324 duplicate studies were excluded, 718 studies that did not meet the inclusion criteria were excluded, and 15 studies were finally included (11–25). The specific process of literature selection is shown in Figure 1.

### 3.2. Basic characteristics of included studies

A total of 15 (11–25) studies were included, of which 11 (11–15, 17–20, 22, 25) were cross-sectional studies and 4 (16, 21, 23, 24) were case–control studies; the highest score of literature quality assessment was 9 (19, 25), and the lowest score was 6 (13). A total of 12,168 study subjects with 18 risk factors were included in the 15 studies. The basic characteristics of the included literature are shown in Table 1.

**Table 1:** Basic characteristics of the included studies.

Study (author/year)	Study type	Province	Total size (*n*)	Influence factors	Quality evaluation
Du 2022 (11)	Cross-sectional study	Tianjin	387	1, 3, 4, 5, 6, 7, 9, 12, 13, 16	8
Liu 2022 (12)	Cross-sectional study	Zhejiang	297	1, 4, 6, 8, 14, 17	8
Chen 2022 (13)	Cross-sectional study	Zhejiang	120	2, 5, 6, 8, 10, 15, 17	6
Yang 2022 (14)	Cross-sectional study	Tianjin	248	5, 6, 8	8
Ye 2022 (15)	Cross-sectional study	Fujian	1,000	1, 2, 6, 8, 9	8
Liu 2022 (16)	Case–control study	Zhejiang	504	6, 7, 11	8
Wang 2021 (17)	Cross-sectional study	Zhejiang	1,300	1, 2, 6, 8	7
Wang 2020 (18)	Cross-sectional study	Zhejiang	296	7	7
Fu 2019 (19)	Cross-sectional study	Hunan	3,513	3, 9, 10	9
Zhao 2016 (20)	Cross-sectional study	Hebei	1,668	1, 2, 6, 18	7
Jia 2014 (21)	Case–control study	Liaoning	298	1, 4, 7, 10, 11, 12, 13	8
Li 2013 (22)	Cross-sectional study	Hebei	590	6, 8, 9, 18	7
Wei 2018 (23)	Case–control study	Gansu	100	1, 4, 10, 11, 12	7
Wei 2019 (24)	Case–control study	Henan	99	2, 11, 14	8
Chu 2022 (25)	Cross-sectional study	Zhejiang	1748	10, 13, 15, 16	9

1, relationship quality; 2, marital status; 3, education level; 4, family income; 5, menstrual status; 6, comorbid chronic diseases; 7, social support; 8, exercise status; 9, perimenopausal syndrome; 10, age; 11, stressful events; 12, quality of life; 13, serum estradiol (E2); 14, BMI; 15, place of residence; 16, FSH (follicle stimulating hormone) level; 17, insomnia; 18, neighbor relationship.

### 3.3. Quality evaluation of studies

Eleven cross-sectional studies (11–15, 17–20, 22, 25) all had quality scores ≥6, and only one (13) had a quality score of 6; four case–control studies (16, 21, 23, 24) all had quality scores ≥7, and the overall quality of the included literature was high.

### 3.4. Meta-analysis results

The number of studies including the education level, relationship with neighbors, BMI, place of residence, insomnia status, and FSH level in this study was limited, so they were not combined for analysis. The results of the meta-analysis showed that relationship quality, marital status (mainly divided into two situations: married and divorced/without spouse), family income, comorbid chronic diseases, social support, exercise status, perimenopausal syndrome, age, and stressful events were associated with perimenopausal depression in Chinese women (*p* < 0.05). The results of the specific analysis are shown in Table 2.

**Table 2:** Meta-analysis of risk factors for perimenopausal depression in Chinese women.

Influencing factor	Studies (*n*)	Heterogeneity test *p* value *I*^2^/%	Effect-model	Meta-analysis results OR (95% CI) *p* value
Relationship quality	6	0.59 0	Fixed-effects	1.23 (1.03, 1.46) 0.02
Marital status	5	0.82 0	Fixed-effects	2.49 (1.77, 3.50) <0.001
Family income	4	0.98 0	Fixed-effects	1.48 (1.10, 2.00) 0.01
Menstrual status	3	0.003 83	Random-effects	2.46 (0.97, 6.25) 0.06
Comorbid chronic diseases	9	<0.001 99	Random-effects	2.39 (1.93, 2.95) <0.001
Social support	4	0.12 49	Fixed-effects	0.76 (0.63, 0.91) 0.004
Exercise status	4	0.47 0	Fixed-effects	1.63 (1.26, 2.11) <0.001
Perimenopausal syndrome	3	0.40 0	Fixed-effects	2.36 (2.11, 2.63) <0.001
Age	4	0.18 38	Fixed-effects	1.04 (1.01, 1.07) 0.006
Stressful events	3	0.29 18	Fixed-effects	12.14 (6.48, 22.72) <0.001
Quality of life	3	0.02 75	Random-effects	0.93 (0.54, 1.60) 0.78
Serum estradiol	3	<0.001 91	Random-effects	1.08 (0.44, 2.65) 0.87

#### 3.4.1. Demographic characteristics

**Figure 2:**
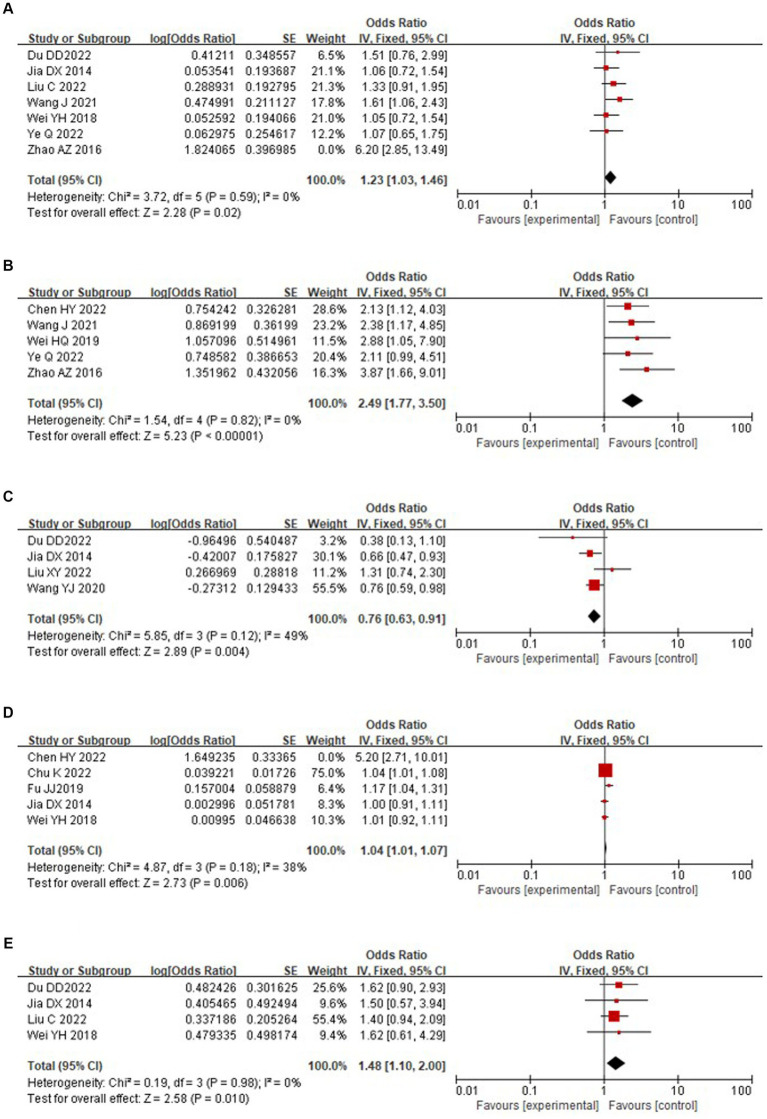
Forest plot of sociodemographic factors of perimenopausal depression in Chinese women. **(A)** Relationship quality. **(B)** Marital status. **(C)** Social support. **(D)** Age. **(E)** Family income.

A total of four factors were included in the analysis, and the results showed that relationship quality, marital status, social support, and age were strongly associated with depression in perimenopausal women. A total of seven studies (11, 12, 15, 17, 20, 21, 23) analyzed the association between relationship quality and depression in perimenopausal women, and the included studies were tested for heterogeneity (*p* = 0.003, *I*^2^ = 69%), and statistical heterogeneity existed among the studies. Sensitivity analysis revealed that the study by Zhao et al. (20) induced heterogeneity and was therefore excluded, and heterogeneity was tested again (*p* = 0.59, *I*^2^ = 0%). A fixed-effects model was selected for the combined analysis, and the results showed a statistically significant difference [OR = 1.23, 95% CI (1.03, 1.46), *p* = 0.02]. A total of five studies (13, 15, 17, 20, 24) analyzed the association between marital status and depression in perimenopausal women, and the included studies were tested for heterogeneity (*p* = 0.82, *I*^2^ = 0%). There was no statistical heterogeneity across studies, and the fixed-effects model was selected for the combined analysis, which showed statistically significant differences [OR = 2.49, 95% CI (1.77. 3.50), *p* < 0.001]. A total of four studies (11, 16, 18, 21) analyzed the association between social support and depression in perimenopausal women, and the included studies were tested for heterogeneity (*p* = 0.12, *I*^2^ = 49%). There was no statistical heterogeneity among the studies, and the results of the combined analysis with a fixed-effects model showed a statistically significant difference [OR = 0.76, 95% CI (0.63. 0.91), *p* = 0.004]. A total of five studies (13, 19, 21, 23, 25) analyzed the association between age and depression in perimenopausal women, and the included studies were tested for heterogeneity (*p* < 0.001, *I*^2^ = 86%). Sensitivity analysis was performed and found that the study by Chen et al. (13) induced heterogeneity and was therefore excluded, and heterogeneity was tested again (*p* = 0.18, *I*^2^ = 38%). A fixed-effects model for combined analysis showed statistically significant differences [OR = 1.04, 95% CI (1.01, 1.07), *p* = 0.006]. The specific analysis is shown in Figure 2.

#### 3.4.2. Socio-economic factor

A total of four studies (11, 12, 21, 23) analyzed the association between family income and depression in perimenopausal women, and the included studies were tested for heterogeneity (*p* = 0.98, *I*^2^ = 0%). There was no statistical heterogeneity among the studies, and the fixed-effects model was selected for the combined analysis, which showed a statistically significant difference [OR = 1.48, 95% CI (1.10, 2.00), *p* = 0.01]. The specific analysis is shown in Figure 2.

#### 3.4.3. Disease and physiological factors

**Figure 3:**
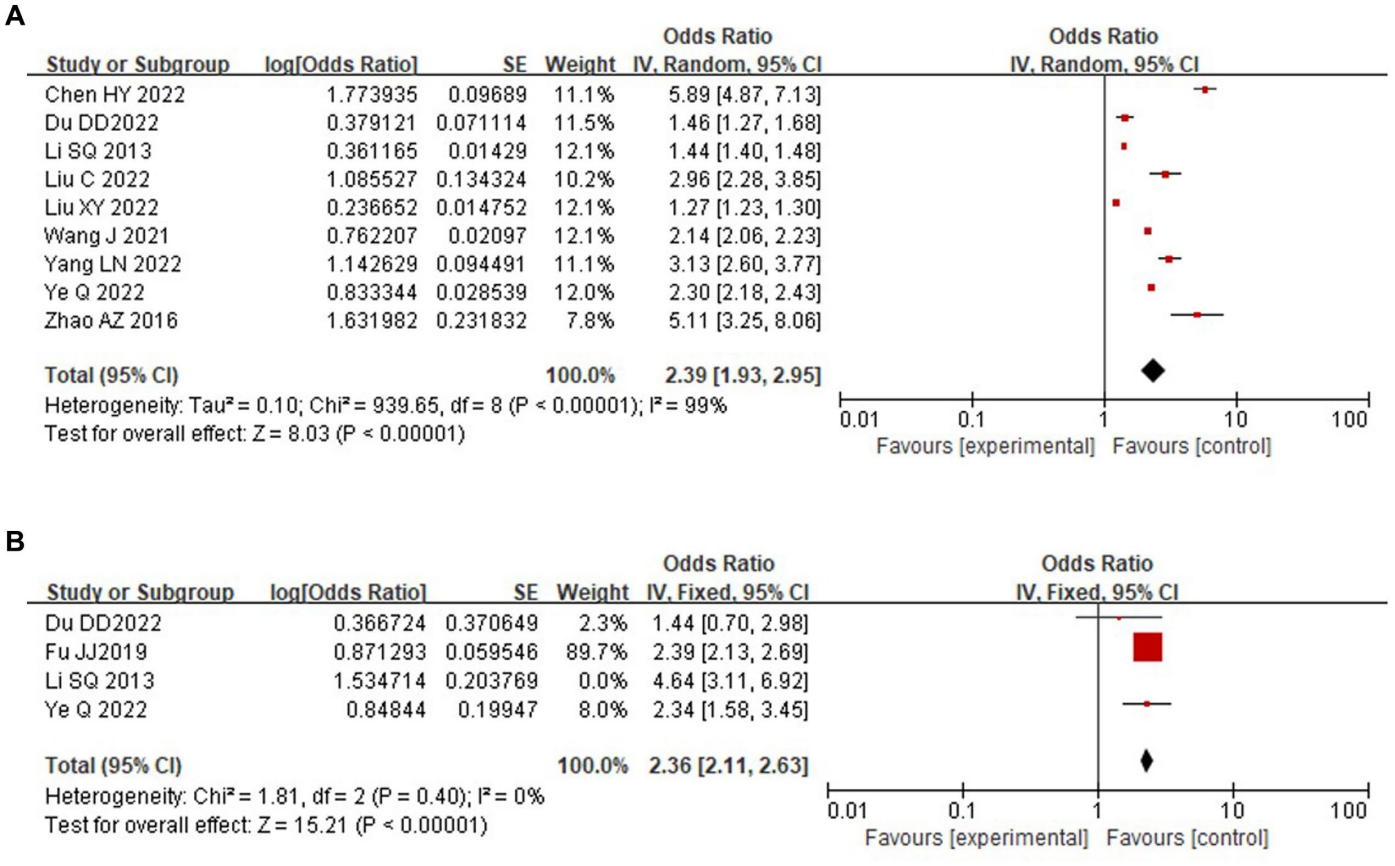
Forest plot of disease factors of perimenopausal depression in Chinese women. **(A)** Comorbid chronic diseases. **(B)** Perimenopausal syndrome.

A total of 2 risk factors were included for analysis, and the results showed that comorbid chronic diseases and perimenopausal syndrome were risk factors for the development of depression in perimenopausal women. A total of nine studies (11–17, 20, 22) analyzed the association between comorbid chronic diseases and depression in perimenopausal women, and the included studies were tested for heterogeneity (*p* < 0.001, *I*^2^ = 99%); statistical heterogeneity existed across studies, and sensitivity analysis revealed that the results of each study were stable, so a random-effects model was chosen for the combined analysis, which showed statistically significant differences [OR = 2.39, 95% CI (1.93, 2.95), *p* < 0.004]. A total of four studies (11, 12, 21, 23) analyzed the association between perimenopausal syndrome and depression in perimenopausal women, and the included studies were tested for heterogeneity (*p* = 0.007, *I*^2^ = 75%). Sensitivity analysis revealed that the study by Li et al. (22) resulted in substantial heterogeneity and was therefore excluded, and heterogeneity was tested again (*p* = 0.40, *I*^2^ = 0%). A fixed-effects model was selected for the combined analysis, and the results showed a statistically significant difference [OR = 2.36, 95% CI (2.11, 2.63), *p* < 0.001]. The specific analysis is shown in Figure 3.

#### 3.4.4. Other factors

**Figure 4:**
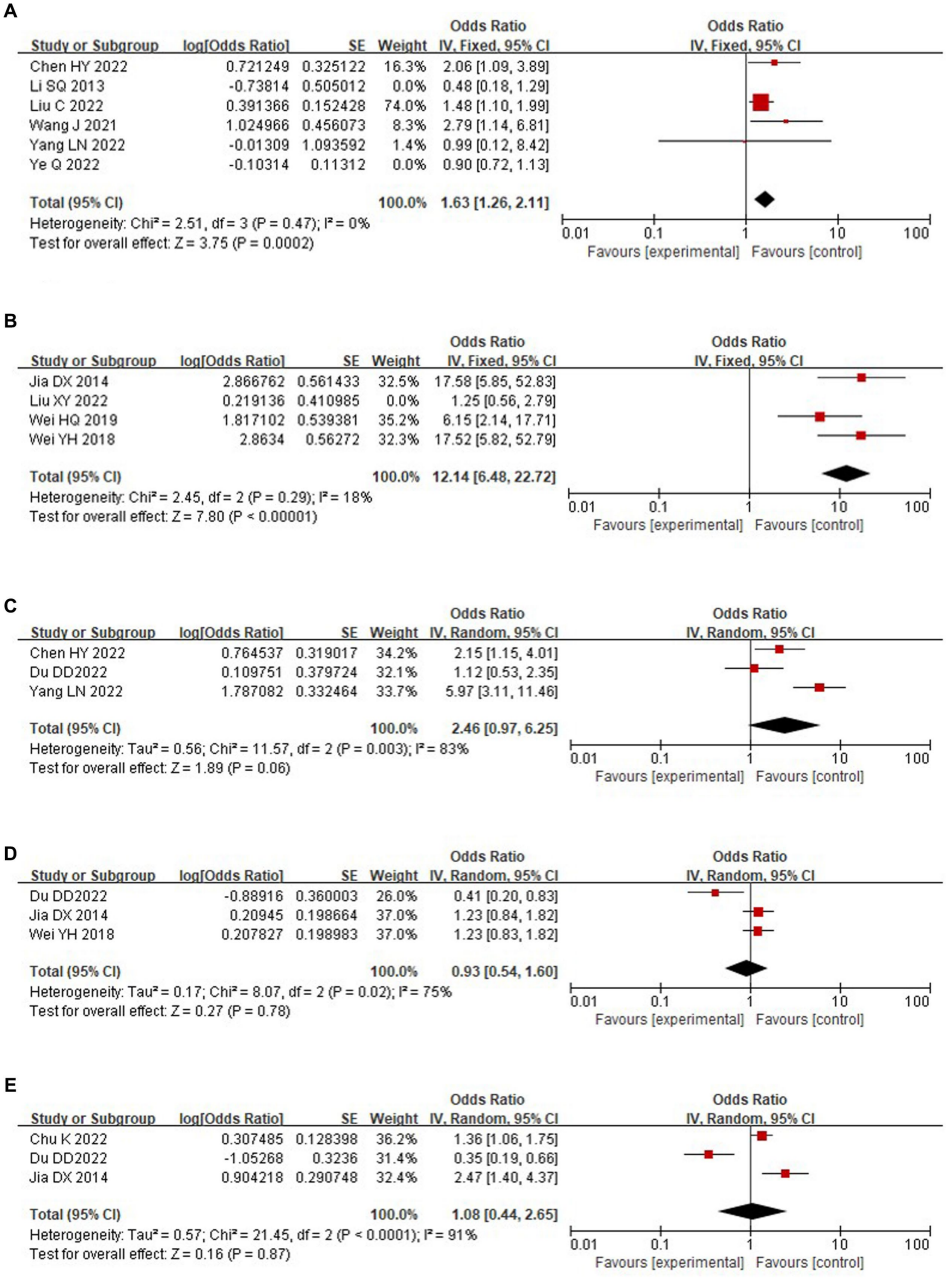
Forest plot of other factors of perimenopausal depression in Chinese women. **(A)** Exercise status. **(B)** Stressful events. **(C)** Menstrual status. **(D)** Quality of life. **(E)** Serum estradiol.

This study analyzed the associations of exercise status, stressful events, menstrual status, quality of life, and serum estradiol with the occurrence of depression in perimenopausal women, and exercise status and stressful events were shown to be strongly associated with the occurrence of depression in perimenopausal women. A total of 6 studies (12–15, 17, 22) analyzed the association between exercise status and depression in perimenopausal women, and the included studies were tested for heterogeneity (*p* = 0.004, *I*^2^ = 71%). Sensitivity analysis revealed large heterogeneity associated with the studies of Ye et al. (15) and Li et al. (22), which were subsequently excluded, and heterogeneity was tested again (*p* = 0.47. *I*^2^ = 0%); a fixed-effects model was selected for the combined analysis, which showed a statistically significant difference [OR = 1.63, 95% CI (1.26, 2.11), *p* < 0.001]. A total of four studies (16, 21, 23, 24) analyzed the association between stressful events and depression in perimenopausal women, and the included studies were tested for heterogeneity (*p* < 0.001, *I*^2^ = 86%). Sensitivity analysis revealed large heterogeneity in the study by Liu et al. (16), which was subsequently excluded, and the heterogeneity test was performed again (*p* = 0.29, *I*^2^ = 18%). A fixed-effects model was used for the combined analysis, and the results were statistically significant [OR = 12.14, 95% CI (6.48, 22.72), *p* < 0.001]. Three studies (11, 13, 14) analyzed the association between menstrual status and depression in perimenopausal women and showed no statistically significant difference [OR = 2.46, 95% CI (0.97, 6.25), *p* = 0.06]. Three studies (11, 21, 23) analyzed the association between quality of life and depression in perimenopausal women and showed no statistically significant difference [OR = 0.93, 95% CI (0.54, 1.60), *p* = 0.78]. Three studies (11, 21, 25) analyzed the association between serum estradiol and depression in perimenopausal women and showed no statistically significant difference [OR = 1.08, 95% CI (0.44, 2.65), *p* = 0.87]. The specific analysis is shown in Figure 4.

### 3.5. Sensitivity analysis

Sensitivity analysis of the research results produced by random-effects and fixed-effects model conversion showed that there was no significant difference in the amount of the combined effect of the two effects models, and the results of the meta-analysis were stable (Table 3).

**Table 3:** Sensitivity analysis for the inclusion of risk factors.

Influence factor	Fixed-effects model OR values 95% CI	Random-effects model OR values 95% CI
Relationship quality	1.23 (1.03, 1.46)	1.23 (1.03, 1.46)
Marital status	2.49 (1.77, 3.50)	2.49 (1.77, 3.50)
Family income	1.48 (1.10, 2.00)	1.48 (1.10, 2.00)
Menstrual status	2.58 (1.75, 3.79)	2.46 (0.97, 6.25)
Combined chronic diseases	1.57 (1.55, 1.60)	2.39 (1.93, 2.95)
Social support	0.76 (0.63, 0.91)	0.77 (0.56, 1.04)
Exercise status	1.63 (1.26, 2.11)	1.63 (1.26, 2.11)
Perimenopausal syndrome	2.36 (2.11, 2.63)	2.36 (2.11, 2.63)
Age	1.04 (1.01, 1.07)	1.04 (0.99, 1.10)
Stressful events	12.14 (6.48, 22.72)	12.18 (6.09, 24.38)
Quality of life	1.07 (0.82, 1.38)	0.93 (0.54, 1.60)
Serum estradiol	1.27 (1.02, 1.57)	1.08 (0.44, 2.65)

## 4. Discussion

### 4.1. Summary of evidence

The results of this study showed that marital status, relationship between husband and wife, social support, family income, and age were strongly associated with depression in perimenopausal women. A study showed that depression is more likely to occur in divorced or unmarried women (26), which may be because when women enter perimenopause, they have different degrees of physiological and psychological problems and need care from their spouses; if they have no spouse, and their children have already started a family, the “empty nest” phenomenon occurs, resulting in a lack individuals to talk to, the accumulation of bad emotions and a greater likelihood of depression. A good or bad relationship between husband and wife has a substantial influence on depression in perimenopausal women, with a poor relationship between husband and wife resulting in emotional instability and increased susceptibility to depression and other negative emotions (11); some studies have shown that the closeness of family relationships is an important factor in the occurrence of depression and even suicide in patients (27). Social support is a protective factor against depression in perimenopausal women; therefore, society, family, and friends should give more care and psychological support to perimenopausal women to help them establish a positive attitude and face that life stage with peace of mind, thus minimizing the incidence of depression (28). Some studies have shown a higher incidence of anxiety and depression in perimenopausal women with a lower annual family income, which may be explained by the fact that perimenopausal women at this stage are not only facing the pressure of having their children leaving to study and start a family but also the obligation to support elderly individuals, which increases psychological stress for women under double economic pressure (29). The same cross-sectional study (30) also confirmed that the older perimenopausal women are, the more likely they are to develop depression, which is consistent with the results of the present study; this may be because perimenopausal women in the late perimenopausal period (>50 years of age) have a significant decline in the physiological function of the ovaries and fluctuations in the levels of a variety of hormones in the body (31), which leads to neurological disorders accompanied by a series of symptoms, such as insomnia, agitation, and emotional instability, and the physiological state and psychological equilibrium of the body has been destroyed, which makes them more prone to develop depression (32). Therefore, it is suggested that professionals in the public health sector conduct risk screening for perimenopausal women who are unmarried, are in poor relationships, have low family income, and are in the late perimenopausal stage (>50 years old) and carry out health education to promote their physical and mental health.

The results of the study showed that comorbid chronic diseases and perimenopausal syndrome are risk factors for depression in perimenopausal women. One study (33) reported that patients with chronic diseases are more prone to depression, which may be because patients with chronic diseases suffer from a variety of illnesses and economic pressures, losing confidence in the treatment and recovery of the disease, coupled with the perimenopausal effects on mood stability, including an increased propensity toward mood fluctuations, all of which results in depression, self-blame, misanthropy and other difficult emotions. Several studies have demonstrated that the more severe the symptoms of perimenopausal syndrome, the greater the chance of depression in perimenopausal women (34, 35); those results may be related to the fact that perimenopausal women experience insomnia, hot flashes, bone and joint pain, emotional distress, and other related symptoms and that living with discomfort can lead to significant mood swings in perimenopausal women, thereby leading to negative emotions and consequently to depression (36). Therefore, it is recommended that perimenopausal women with chronic diseases should increase the treatment and prevention of chronic diseases to alleviate the somatic pain and physical damage caused by chronic diseases and reduce the development of negative emotions; female patients with perimenopausal syndrome can be treated under the guidance of a professional doctor to relieve physical discomfort, maintain a healthy diet and a happy mood in daily life, and improve life satisfaction, thus preventing the occurrence of depression.

The results of this study showed that exercise status and stressful events were strongly associated with the occurrence of depression in perimenopausal women. The study demonstrated that perimenopausal women who lacked exercise were more likely to experience depression than perimenopausal women who exercised regularly (37), which may be because during exercise, the body releases endogenous peptides that can produce sustained pleasure and that during exercise, women’s stress is released and emotions are transferred, thus reducing the occurrence of depression. Therefore, it is recommended that perimenopausal women adhere to exercise programs to increase basal metabolic levels, improve sleep quality, and relieve physical and mental stress, thus maintaining emotional stability. Studies have also shown that perimenopausal women who experience stressful events have a significantly higher risk of depression than perimenopausal women who do not experience stressful events (24). Perimenopausal women face pressure from various aspects, and stressful events occur frequently in their lives. Therefore, it is recommended that perimenopausal women participate in more activities to improve their state of mind, correctly recognize the occurrence of objective events, use reasonable methods to alleviate adverse emotions, and try to avoid depression due to stressful events.

### 4.2. Advantages and limitations

The present study has many advantages. First, we only included relevant factors for analysis in the multifactor logistic regression analysis, which reduced the generation of confounding bias to a certain extent. Second, the quality of the included literature in this study was generally high, and the whole meta-analysis process strictly followed PRISMA reporting norms, which made the study results more reliable. Finally, this study is the first systematic analysis of risk factors for perimenopausal depression in Chinese women, oriented to clinical problems, with the aim of providing a basis for screening high-risk groups of perimenopausal women for depression. However, there are some shortcomings in this study. First, the associations of menstrual status, quality of life, and serum estradiol with the occurrence of depression in perimenopausal women could not be determined in this study, which may be related to the small number of included studies and the individual differences in the included subjects. Second, the 12 risk factors analyzed in this study were not tested for publication bias due to the limited number of studies included in each; therefore, publication bias may exist.

### 4.3. Clinical significance

The results of this study show that depression in Chinese perimenopausal women is influenced by multiple factors. The public health sector can carry out health literacy about the risk factors for depression in perimenopausal women, such as informing perimenopausal women that marital disharmony, absence of spouse, lower family income, comorbidity with chronic diseases, presence of perimenopausal syndromes, stressful events, lack of physical activity, and late perimenopausal period (age > 50 years) are the risk factors for perimenopausal women’s depression, to increase awareness of prevention among perimenopausal women and to reduce the global incidence of depression in perimenopausal women.

## 5. Conclusion

In this study, we systematically searched for studies related to perimenopausal depression in Chinese women and strictly followed the meta-analysis production process to summarize and analyze their influencing factors. It was concluded that poor marital relationships, no spouse, low family income, comorbid chronic diseases, lack of exercise, perimenopausal syndrome, late perimenopausal period, and stressful events were risk factors for depression in perimenopausal women, while social support was a protective factor against depression in perimenopausal women; this provides a reference basis for global preventive health care for the occurrence of depression in perimenopausal women. However, the subjects included in this study were mostly from China, which may affect the comprehensiveness of the results. In the future, more high-quality global studies on risk factors for depression in perimenopausal women should be included to obtain more comprehensive results.

## Data availability statement

The original contributions presented in the study are included in the article/Supplementary material, further inquiries can be directed to the corresponding author.

## Author contributions

QG: conceptualization, study design, literature search, quality evaluation, writing the manuscript. RY: study design, literature search, quality evaluation. ZL: data extraction, methodological guidance, proofreading of data. LW: data extraction, data proofreading. YL: organizing data, proofreading data. YY: organizing data, proofreading data. LZ: supervision, financial support, revision of manuscripts. All authors contributed to the article and approved the submitted version.

## Footnotes

^1^ frontiersin.org
