# Supplementary material for: Risk factors for perimenopausal depression in Chinese women: a meta-analysis
Source: Front Psychiatry. 2023 Oct 11;14:1199806. doi: 10.3389/fpsyt.2023.1199806 (PMC10598844; doi:10.3389/fpsyt.2023.1199806)
Supplement: Supplementary file 1 [file Data_Sheet_1.docx]

Supplementary Material

Risk factors for perimenopausal depression in Chinese women: A meta-analysis

Qingwen Gan^1, 2^, Ran Yu^1, 2^, Zerong Lian^3^, Lihua Wei^1^, Yuanping Li^1^, Yiling Yuan^1^, and Lilan Zheng^2^*

^1^School of Nursing, Nanchang University, Nanchang 330006, China.

^2^The First Affiliated Hospital of Nanchang University, Nanchang 330006, China.

^3^Heping Hospital Affiliated to Changzhi Medical College, Changzhi 046000, China.

* Corresponding author: Lilan Zheng
E-mail: [2737424497@qq.com](mailto:2737424497@qq.com)

**English Database Retrieval Strategy**

**1 PubMed (164)**

#1 "Menopause"[MeSH Terms]

#2 (((((Perimenopause[Title/Abstract]) OR (climacteric[Title/Abstract])) OR (Change of Life, Female[Title/Abstract])) OR (perimenopausal syndrome[Title/Abstract])) OR (menopausal syndrome[Title/Abstract])) OR (climacteric syndrome[Title/Abstract])

#3 #1 OR #2

#4 "Depression"[MeSH Terms]

#5 "depressive symptoms"[Title/Abstract] OR "depressive symptom"[Title/Abstract] OR "symptom depressive"[Title/Abstract] OR "emotional depression"[Title/Abstract] OR "depression emotional"[Title/Abstract] OR "depressed"[Title/Abstract]

#6 #4 OR #5

#7 "Risk Factors"[MeSH Terms]

#8 "risk factors"[Title/Abstract] OR "related factors"[Title/Abstract] OR "influence factor"[Title/Abstract] OR "factor risk"[Title/Abstract] OR "risk factor"[Title/Abstract]

#9 #7 OR #8

#10 #3 AND #6 AND #9

**2 Web of science (10)**

#1 risk factors (Title) or related factors (Title) or influence factor (Title) or factor risk (Title) or risk factor (Title)

#2 climacteric syndrome (Title) or Perimenopause (Title) or Menopause (Title) or climacteric (Title) or perimenopausal syndrome (Title) or menopausal syndrome (Title) or Change of Life, Female (Title)

#3 Depression (Title) or depressive symptoms (Title) or depressive symptom (Title) or symptom depressive (Title) or emotional depression (Title) or depression emotional (Title) or depressed (Title)

#4 #1 AND #2 AND #3

**3 The Cochrane library (113)**

#1 (risk factors):ti,ab,kw OR (related factors):ti,ab,kw OR (influence factor):ti,ab,kw OR (factor risk):ti,ab,kw OR (risk factor):ti,ab,kw

#2 (Depression):ti,ab,kw OR (depressive symptoms):ti,ab,kw OR (depressive symptom):ti,ab,kw OR (symptom depressive):ti,ab,kw OR (depressed):ti,ab,kw

#3 (climacteric syndrome):ti,ab,kw OR (Menopause):ti,ab,kw OR (Perimenopause):ti,ab,kw OR (perimenopausal syndrome):ti,ab,kw OR (menopausal)

#4 #1 AND #2 AND #3

**4 知网（CNKI 311）**

#1 影响因素 + 相关因素 + 危险因素 + 风险因素 + 预测因素 + 原因

#2 围绝经期综合征 + 绝经期综合征 + 绝经期 + 更年期 + 更年期综合征 + 妇女更年期综合征 + 妇更征

#3 抑郁 + 抑郁症

#4 #1 AND #2 AND #3

**5 万方（ Wanfang 158）**

#1 影响因素 OR 相关因素 OR 危险因素 OR 风险因素 OR 预测因素 OR 原因

#2 围绝经期综合征 OR 绝经期综合征 OR 绝经期 OR 更年期 OR 更年期综合征 OR 妇女更年期综合征 OR 妇更征

#3 抑郁 OR 抑郁症

#4 #1 AND #2 AND #3

**6 维普(VIP 109)**

#1 影响因素 OR 相关因素 OR 危险因素 OR 风险因素 OR 预测因素 OR 原因

#2 围绝经期综合征 OR 绝经期综合征 OR 绝经期 OR 更年期 OR 更年期综合征 OR 妇女更年期综合征 OR 妇更征

#3 抑郁 OR 抑郁症

#4 #1 AND #2 AND #3

**7 Sinomed (192)**

#1 "抑郁"[不加权:扩展]

#2 "抑郁症"[不加权:扩展]

#3 ("抑郁症"[不加权:扩展]) OR ("抑郁"[不加权:扩展])

#4 "影响因素"[常用字段:智能] OR "相关因素"[常用字段:智能] OR "危险因素"[常用字段:智能] OR "风险因素"[常用字段:智能] OR "预测因素"[常用字段:智能] OR "原因"[常用字段:智能]

#5 "围绝经期综合征"[常用字段:智能] OR "绝经期综合征"[常用字段:智能] OR "绝经期"[常用字段:智能] OR "更年期"[常用字段:智能] OR "更年期综合征"[常用字段:智能] OR "妇女更年期综合征"[常用字段:智能] OR "妇更征"[常用字段:智能]

#6 #3 AND #4 AND #5
